# Practical guidance on the use of sacubitril/valsartan for heart failure

**DOI:** 10.1007/s10741-018-9757-1

**Published:** 2018-12-18

**Authors:** Andrew J. Sauer, Robert Cole, Brian C. Jensen, Jay Pal, Nakul Sharma, Amin Yehya, Justin Vader

**Affiliations:** 1 Center for Advanced Heart Failure and Heart Transplantation, The University of Kansas Health System, 3901 Rainbow Boulevard Mailstop 1072, Kansas City, KS 66160 USA; 2 Center for Heart Failure Therapy and Transplantation, Emory University, Atlanta, GA USA; 3 UNC McAllister Heart Institute, The University of North Carolina at Chapel Hill, Chapel Hill, NC USA; 4 Division of Cardiothoracic Surgery, University of Colorado, Aurora, CO USA; 5 Libin Cardiovascular Institute of Alberta, Cummings School of Medicine, University of Calgary, Calgary, Alberta Canada; 6 Advanced Heart Failure and Heart Transplant, Piedmont Heart Institute, Atlanta, GA USA; 7 Department of Medicine, Division of Cardiology, Washington University in St Louis, St Louis, MO USA

**Keywords:** Heart failure, Sacubitril/valsartan, Cardiovascular, Angiotensin receptor-neprilysin inhibitor, Reduced ejection fraction

## Abstract

Sacubitril/valsartan is a first-in-class angiotensin receptor-neprilysin inhibitor (ARNI) that has been recommended in clinical practice guidelines to reduce morbidity and mortality in patients with chronic, symptomatic heart failure (HF) with reduced ejection fraction (HFrEF). This review provides an overview of ARNI therapy, proposes strategies to improve the implementation of sacubitril/valsartan in clinical practice, and provides clinicians with evidence-based, practical guidance on the use of sacubitril/valsartan in patients with HFrEF. Despite evidence demonstrating the benefits of ARNI therapy over standard of care, only a fraction of eligible patients takes sacubitril/valsartan. Barriers preventing the prescription of sacubitril/valsartan in eligible patients may include practitioners’ unfamiliarity with ARNIs, safety concerns, and payer reimbursement issues. The optimal implementation of sacubitril/valsartan in clinical practice has the potential to reduce the overall burden of HF. Throughout this review, we describe our experience with sacubitril/valsartan, including strategies for the management of adverse events and common patient concerns. In addition, a strategy for the gradual introduction of sacubitril/valsartan using a treatment sequence scheme is proposed.

## Introduction

**Fig. 1:**
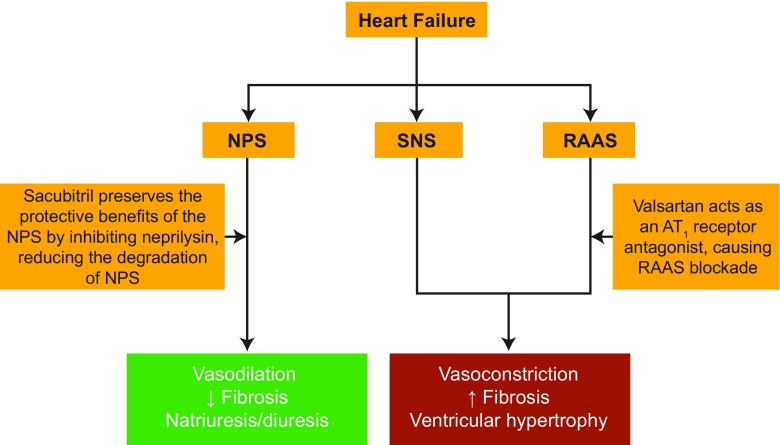
Mechanism of action of sacubitril/valsartan [5]. Adapted with permission from [6]. Abbreviations: *AT*_1_ angiotensin type 1, *NP* natriuretic peptide, *NPS* natriuretic peptide system, *RAAS* renin–angiotensin–aldosterone system, *SNS* sympathetic nervous system

Approximately 60,000 US deaths per year are attributed to heart failure (HF) [1]. However, few pharmacological classes can reduce HF mortality. Sacubitril/valsartan [2, 3], a first-in-class angiotensin receptor-neprilysin inhibitor (ARNI), contains the angiotensin receptor blocker (ARB) valsartan and a neprilysin inhibitor prodrug, sacubitril (AHU377), which is metabolized to the active metabolite, LBQ657 [4]. This drug targets two pathways critical for HF pathobiology (Fig. 1). ARNIs may present significant advancement over angiotensin-converting enzyme (ACE) inhibition or angiotensin receptor blockade alone, because neprilysin inhibition acts synergistically with renin–angiotensin–aldosterone system (RAAS) blockade to prevent cardiac remodeling and support cardiomyocyte survival [7].

In PARADIGM-HF (Prospective Comparison of ARNI with ACEI [ACE inhibitor] to Determine Impact on Global Mortality and Morbidity in Heart Failure; NCT01035255), a randomized, double-blind (DB), parallel-group study, the synergistic effects of RAAS and neprilysin inhibition (sacubitril/valsartan) versus RAAS inhibition alone (enalapril) led to significantly lower all-cause and cardiovascular mortality for HF with reduced ejection fraction (HFrEF) [8]. PARADIGM-HF was one of the largest clinical trials ever conducted in HF (*N* = 8442) and was the pivotal phase 3 trial that led to Food and Drug Administration (FDA) approval of sacubitril/valsartan [8–10].

PARADIGM-HF was stopped early (median follow-up 27 months) because sacubitril/valsartan met the prespecified primary endpoint of showing superiority to enalapril for reducing the rate of cardiovascular death or hospitalizations for HF (hazard ratio (HR) 0.80; 95% confidence interval (CI) 0.73–0.87; *P* < 0.001) [8]. Benefits were also reported for cardiovascular death (HR 0.80; 95% CI 0.71–0.89; *P* < 0.001) and first hospitalization for worsening HF (HR 0.79; 95% CI 0.71–0.89; *P <* 0.001) [8]. Death from any cause was also 16% lower with sacubitril/valsartan versus enalapril (95% CI 0.76–0.93; *P* < 0.001) [8].

Calculation of number-needed-to-treat (NNT) from PARADIGM-HF demonstrated that 21 patients needed treatment with sacubitril/valsartan instead of enalapril for 27 months to prevent one death from a cardiovascular cause or hospitalization for HF [8]. These NNTs were applied to an estimate of the current number of US patients with HFrEF (based on data from the 2016 American Heart Association Heart Disease and Stroke Statistics Update) who were candidates for therapy, excluding patients receiving hospice care or advanced HF management [11]. Accordingly, ~ 28,484 deaths (range 18,230–41,017) could be prevented each year with optimal implementation of ARNI therapy instead of ACEIs/ARBs [11]. The potential to prevent > 20,000 deaths favors accelerated clinical implementation of ARNIs [11].

The 2017 update of the American College of Cardiology/American Heart Association/Heart Failure Society of America (ACC/AHA/HFSA) guideline for HF management included ARNIs, along with ACEIs and ARBs, as a treatment to reduce mortality and morbidity in HFrEF [3, 11]. The guideline recommends (class I recommendation, moderate-quality evidence) replacing ACEIs or ARBs with an ARNI in chronic, symptomatic, or New York Heart Association (NYHA) class II or III HFrEF to further reduce morbidity and mortality, provided there are no contraindications (i.e., history of angioedema or hypersensitivity to any drug component) [2, 3]. However, ~ 10% of 2.29 million eligible patients use sacubitril/valsartan [11, 12]. Barriers to clinical implementation may include clinician unfamiliarity, reluctance to switch stable patients, safety concerns, and payer-reimbursement issues [12–15]. Although these concerns are common with newly approved drugs, delays in prescribing sacubitril/valsartan could have significant impact on public health [11]. This article addresses these concerns by reviewing the efficacy and safety of sacubitril/valsartan based on its unique mechanism of action (MOA), proposing potential solutions to barriers of implementing ARNIs, and providing evidence-based guidance on sacubitril/valsartan use in HFrEF. Throughout this article, we also describe our clinical experience with sacubitril/valsartan and our management of adverse events (AEs), including potential questions and concerns posed by patients.

## Practical experience

The benefits of sacubitril/valsartan in PARADIGM-HF are substantiated by real-world studies. In a retrospective cohort study of HFrEF (*N* = 132), reduced risks of mortality at 6 months (OR 0.14; 95% CI 0.04–0.50) and HF hospitalization (OR 0.03; 95% CI 0.01–0.14) were observed with sacubitril/valsartan versus conventional therapy [16]. Safety outcomes were comparable between groups, although sacubitril/valsartan correlated with higher risk of hypotension versus conventional therapy (OR 3.14; 95% CI 0.94–10.55) [16]. Our clinical experiences with treatment response and AE profile for sacubitril/valsartan are consistent with these real-world results and PARADIGM-HF. Thus, clinicians must monitor for hypotension, dizziness, cough, angioedema, hyperkalemia, and renal dysfunction to prevent serious AEs [10]. With an AE profile similar to that of ACEIs/ARBs (Table 1), satisfactory tolerance to sacubitril/valsartan is expected.

**Table 1:** Common AEs (≥ 2%) in the PARADIGM-HF trial during the double-blind treatment period [8]

Preferred term, *n* (%)	Sacubitril/valsartan, *n* = 4203	Enalapril, *n* = 4229	Total, *N* = 8432
At least 1 AE	3419 (81.35)	3503 (82.83)	6922 (82.09)
Hypotension	740 (17.61)	506 (11.97)	1246 (14.78)
Cardiac failure	730 (17.37)	832 (19.67)	1562 (18.52)
Hyperkalemia	488 (11.61)	592 (14.00)	1080 (12.81)
Renal impairment	426 (10.14)	487 (11.52)	913 (10.83)
Cough	369 (8.78)	533 (12.60)	902 (10.70)
Dizziness	266 (6.33)	206 (4.87)	472 (5.60)
Atrial fibrillation	251 (5.97)	236 (5.58)	487 (5.78)
Pneumonia	227 (5.40)	237 (5.60)	464 (5.50)
Peripheral edema	215 (5.12)	213 (5.04)	428 (5.08)

Used with permission from [8]. Copyright 2014: Massachusetts Medical Society

*AE* adverse event, *PARADIGM-HF* Prospective Comparison of ARNI With ACEI to Determine Impact on Global Mortality and Morbidity in Heart Failure

Another retrospective cohort study of 48 patients with HFrEF treated with sacubitril/valsartan observed a reverse remodeling effect after 3 months, as assessed by echocardiographic variables [17]. With sacubitril/valsartan therapy, left ventricular (LV) ejection fraction (EF) increased significantly from 25.33% at baseline to 30.14% at follow-up (*P* < 0.001) [17]. These results are also consistent with our clinical experience, in which patients have demonstrated improvements in EF and reductions in LV size (reversal of LV remodeling). Consequently, these functional changes reduce HF symptoms, reflected by improvement in NYHA class IIIb to class II/I. Case studies of sacubitril/valsartan have similarly observed reversal of LV remodeling and improvements in NYHA class [18–20]. Analysis of health-related quality of life (QOL) in PARADIGM-HF observed that sacubitril/valsartan had a more favorable effect on Kansas City Cardiomyopathy Questionnaire scores versus enalapril at 8 months [21].

In our experience and the literature, when medications improve QOL, adherence improves [22]. This is especially important in chronic diseases for which patients must take medicines indefinitely. Adherence to the prescribed regimen is vital for continued efficacy, yet studies suggest 30–50% of HF medications are not taken as prescribed [23–25]. In our practices, adherence with sacubitril/valsartan appears high in patients who tolerate it.

## Primary barriers to implementation

Cardiologists have considerable reluctance to transition patients stable on current therapy, despite the findings of PARADIGM-HF [12]. Although the controlled conditions of clinical trials support internal validity of safety and efficacy results, lack of real-world evidence when a new drug is approved limits the translation of conclusions to patient populations encountered in clinical practice. Understandably, publications on new drugs are written from a research perspective and may not apply to all patients [26]. Many clinicians will not prescribe new medications until additional real-world data become available [27].

Similarly, clinicians may fear the hypothetical long-term effects of sacubitril/valsartan therapy, such as cognitive impairment due to the inhibition of β-amyloid degradation by sacubitril [28]. In healthy volunteers, no increase in β-amyloid concentration in cerebrospinal fluid was found [29], and results from PARADIGM-HF showed no increase in cognitive defects with sacubitril/valsartan compared with enalapril [30]. The Efficacy and Safety of LCZ696 Compared to Valsartan on Cognitive Function in Patients With Chronic Heart Failure and Preserved Ejection Fraction (PERSPECTIVE; NCT02884206) trial will collect data on the long-term cognitive effects of sacubitril/valsartan [28].

Some clinicians may lack confidence in identifying the appropriate sacubitril/valsartan patient population in clinical practice for fear of causing worsening symptoms in both stable and unstable patients. They may also be unfamiliar with potential side effects that occur during treatment initiation or uptitration and may not know how to mitigate or manage these risks [14]. Implementing a new drug into clinical practice is challenging, even for experienced clinicians.

## Overcoming barriers to implementation

**Fig. 2:**
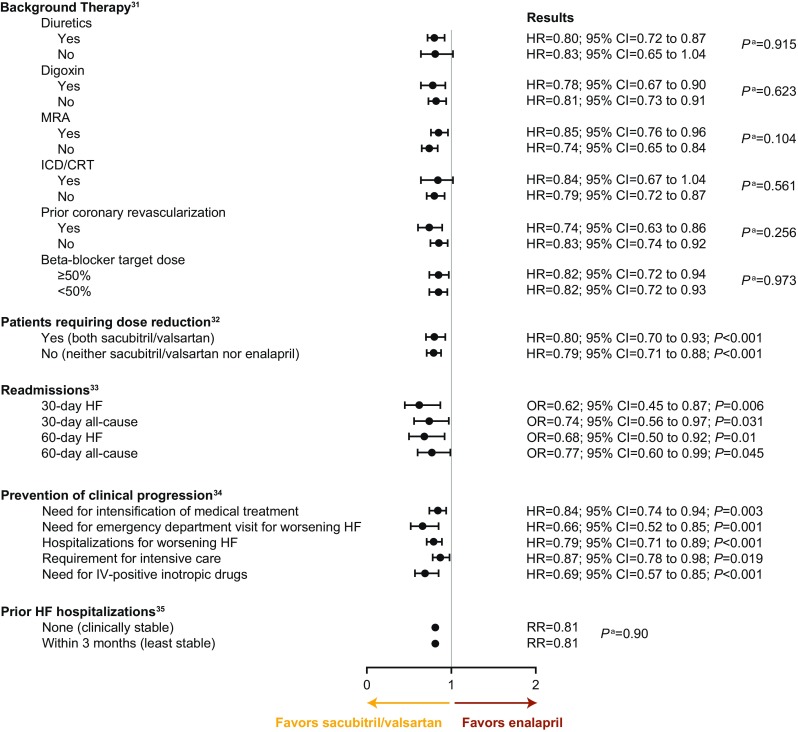
Sacubitril/valsartan vs enalapril: post hoc subanalyses of the PARADIGM-HF trial’s primary endpoint (composite of death from cardiovascular causes or hospitalization for heart failure). ^a^Interaction *P* value. Abbreviations: *CI* confidence interval, *CRT* cardiac resynchronization therapy, *HF* heart failure, *HR* hazard ratio, *ICD* implantable cardioverter–defibrillator, *IV* intravenous, *MRA* mineralocorticoid receptor antagonist, *OR* odds ratio, *RR* relative risk

Subgroup analyses from PARADIGM-HF support efficacy of sacubitril/valsartan regardless of background therapy, clinical stability, or dose reductions (Fig. 2) [31–35], and may reassure clinicians about the drug’s effectiveness in broad patient populations. Besides subgroup analyses, supplementary analyses of data from PARADIGM-HF have modeled the clinical benefits of sacubitril/valsartan beyond measured endpoints [33, 34, 36]. When trial data were used to generate actuarial estimates of outcomes for long-term treatment, the predicted benefit of sacubitril/valsartan rather than enalapril for a patient aged ≥ 55 years was an additional 2.1 years free of cardiovascular-related death or hospitalization; for a patient aged ≥ 65 years, the predicted benefit was 1.6 years [36]. Information on pharmacoeconomics and experience addressing payer concerns can be as important to the clinical implementation of a new therapy as therapeutic data and experience. Recent cost-effectiveness analyses of sacubitril/valsartan support its use [37–39]. Insurance coverage has also improved considerably, with an estimated 90% of eligible patients having coverage [40].

By disseminating new information and sharing their clinical experiences with sacubitril/valsartan, clinicians can help colleagues make informed treatment decisions and ensure that patients obtain optimal care. Several measures can facilitate implementation and encourage acceptance of this novel therapy. Practice sites may provide nurses with training regarding the paperwork and requirements for sacubitril/valsartan reimbursement (e.g., preauthorization). Appropriately documenting the rationale for the treatment plan may facilitate payer authorization, including documenting individual NYHA functional classification, HF symptoms, and blood pressure (BP).

## Initiating therapy

### Patient selection

It may be unexpectedly challenging to identify patients most likely to derive survival and QOL benefits from sacubitril/valsartan. Considering the characteristics of patients in PARADIGM-HF is useful for predicting how real-world patients may respond to therapy. However, it may be difficult to accurately assess patients in clinical practice. For example, patients with chronic HF typically learn to avoid elective activities that may cause dyspnea or excessive fatigue, thereby redefining a new baseline QOL [41, 42]. Thus, these patients may report that they are fine and never have dyspnea, leading them to appear completely or nearly asymptomatic. It is useful to take extra time during routine visits to identify patients who are nearly or completely asymptomatic because of reduced activity.

Ambulatory patients with HFrEF but improved EF of > 35% or those with no indicators of volume overload or symptoms may be less ill than the PARADIGM-HF cohort. Given the known AEs of sacubitril/valsartan, patients need to be screened for hypotension, dizziness, hyperkalemia, renal impairment, cough, and angioedema [10]. Recalling the differences in side-effect profiles between sacubitril/valsartan and enalapril in PARADIGM-HF can help clinicians select patients most likely to tolerate the switch to sacubitril/valsartan.

Although most AEs occurred at a similar rate (Table 1), more patients taking sacubitril/valsartan developed symptomatic hypotension versus those taking enalapril. Thus, risk factors for hypotension should be carefully assessed and proactively managed before initiating therapy [8, 10]. Careful assessment of hypotension is especially important in patients with marginal BP whose degree of decompensation differs from those in PARADIGM-HF.

In PARADIGM-HF, angioedema risk was similar for sacubitril/valsartan and enalapril; however, rates trended higher with sacubitril/valsartan (0.5% vs 0.2%; *P* = 0.13) [8]. The risk of angioedema with sacubitril/valsartan is less than that of previous neprilysin inhibitors (e.g., omapatrilat; 2.17%) [8, 43]. However, patients with a history of angioedema should not be initiated on sacubitril/valsartan because of the complication risk; these patients were excluded from PARADIGM-HF [8]. Because PARADIGM-HF suggests that black patients have a higher rate of angioedema with sacubitril/valsartan versus enalapril (2.4% vs 0.5%, respectively), they should be appropriately counseled about angioedema risk and maintaining vigilance regarding symptoms [10]. Notably, black patients did receive similar benefits compared with the overall study population from sacubitril/valsartan therapy.

Another patient characteristic to consider is patient age. Because 1.4% of patients enrolled in PARADIGM-HF were ≥ 85 years old, the benefits observed from sacubitril/valsartan therapy were not statistically significant in this subpopulation [28, 44]. Furthermore, elderly patients may have multiple comorbidities that could limit dose titration and reduce the benefits of sacubitril/valsartan therapy [28]. However, it is important to note that there is very little trial data for the use of other HF medications in the elderly, and that the PARADIGM-HF trial did include elderly patients ≥ 75 years old, comprising 18.6% of the total trial population [44]. Data from real-world registries will provide more insight into this potential issue [28].

Practitioners should be aware of potential selection bias due to the run-in periods of PARADIGM-HF [8]. Until additional real-world data are available, AEs with sacubitril/valsartan from the trial should be viewed conservatively as being potentially the minimum rate of AE occurrence. With proper preparation prior to initiating therapy and regular monitoring, side effects associated with sacubitril/valsartan can be successfully managed.

### Initial dose selection and uptitration

Sacubitril/valsartan should be used as a replacement for existing ACEI/ARB medication, instead of as an additional therapy. This was typically viewed favorably by our patients. In our experience, the 36-h washout period required when switching therapies requires some logistical planning to ensure patients do not continue ACEI/ARB therapy. One approach is to instruct the patient to throw away the ACEI/ARB medication. If the patient is not comfortable with this approach, the patient can tape prescription bottles closed and safely store them.

When switching from ACEI/ARB to ARNI therapy, the initial dose of sacubitril/valsartan should be similar to the currently prescribed regimen [8, 10, 45]. Patients on low-dose enalapril should be initiated on a low dose of sacubitril/valsartan (24/26 mg twice daily) [10]. Subsequently, uptitration should occur every 2–4 weeks, as tolerated, to the target dose of sacubitril/valsartan (97/103 mg twice daily) [8, 10].

A DB randomized trial observed that ACEI/ARB-naive patients and those who switched from lower-dose ACEI/ARB therapy achieved higher rates of treatment success from uptitration over 6 weeks versus for 3 weeks (84.9% vs 73.6%, respectively; *P* = 0.030) [45]. A more conservative uptitration approach may be considered if tolerance is a concern, particularly with renal impairment or hypotension.

### Preparing for AEs

Clinicians should adequately inform patients of possible AEs when beginning a new medication. Common patient questions and concerns are listed in Table 2. Many patients have received an ACEI/ARB regimen, so they will be familiar with the risks for some AEs. All patients should be prepared for hypotension and orthostatic symptoms [10], the most common symptomatic AEs in PARADIGM-HF [8]. Clinicians must advise patients to take the risk of these potential AEs seriously [10] and make an effort to prevent symptoms by avoiding dehydration, transitioning slowly from standing and sitting, and monitoring weight and BP daily. To prevent hypotension and potential consequent hospitalizations in patients taking diuretics, clinicians may need to reduce the dose or be proactive by discontinuing the medication. Notably, treatment with sacubitril/valsartan may reduce the need for loop diuretic therapy. In PARADIGM-HF, sacubitril/valsartan was associated with fewer loop diuretic dose increases and more dose decreases versus enalapril at 6, 12, and 24 months (net increase in diuretic use for sacubitril/valsartan vs enalapril, respectively; at 6 months, 0.8% vs 2.5%, *P* = 0.05; at 12 months, 1.0% vs 4.6%, *P* < 0.001; at 24 months, 1.9% vs 6.9%, *P* < 0.001) [46]. Correspondingly, hypotension may be prevented by discontinuation or down-titration of other potentially contributory medications (e.g., calcium channel blockers), because they are associated with less evidence-based morbidity and mortality benefits [45, 47]. Although discontinuations due to AEs were significantly lower with sacubitril/valsartan versus enalapril (10.7% vs 12.3%, respectively; *P* = 0.03) [8], taking these precautions will help prevent serious AEs and subsequent therapy discontinuation.

**Table 2:** Common patient questions and concerns regarding sacubitril/valsartan

Questions regarding the benefits of sacubitril/valsartan • I have been stable on my current medications. Why would I change? • How will this medication help my heart? What is the purpose of increasing the dose if I am feeling good on this dose and my blood pressure is good? • Why do I have to be on this medication if my blood pressure is not high? Is this medication for blood pressure? • How long will I live if I take all my medications and monitor what I eat and drink? • Will my heart function recover on medications? • Can I come off some medications if my heart function improves? Do I need to take these medications for the rest of my life? • I am trying to avoid an implantable cardioverter–defibrillator. Will this help? Will this medication improve my ejection fraction? Questions regarding possible negative effects of sacubitril/valsartan • I take so many medications. Will they interact with each other and cause harm? • What are the side effects of medications? • If I do not tolerate the medications, what options do I have? Questions regarding the cost of sacubitril/valsartan • Will my insurance pay for the new medications? • How much more does it cost? Other questions/concerns regarding sacubitril/valsartan • Why have my other doctors not mentioned it? • I am afraid to switch to a medication that is not commonly used.	

Laboratory testing should be performed periodically to monitor for significant clinical changes, including serum potassium and estimated glomerular filtration rate (eGFR) [10]. Because the steady state of sacubitril/valsartan is reached 3 days after initiating therapy [4, 10], we suggest performing laboratory tests then. Although sacubitril/valsartan is associated with electrolyte abnormalities, the incidence of hyperkalemia was lower with sacubitril/valsartan versus enalapril (11.6% vs 14.0%, respectively) during the DB treatment period in PARADIGM-HF [8]. Correspondingly, higher rates of severe hyperkalemia were observed with enalapril versus sacubitril/valsartan among patients treated with mineralocorticoid receptor antagonist (MRA) therapy at baseline (3.1 vs 2.2 per 100 patient-years; HR 1.37; 95% CI 1.06–1.76; *P* = 0.02) and those who were initiated on MRA therapy during the trial (3.3 vs 2.3 per 100 patient-years; HR 1.43; 95% CI 1.13–1.81; *P* = 0.003) [48]. These results suggest that sacubitril/valsartan may attenuate hyperkalemia risk associated with MRA [48].

Occurrence of serious AEs, including angioedema or shock, should prompt permanent discontinuation of therapy. Overall, discontinuations due to AEs were significantly lower with sacubitril/valsartan versus enalapril (10.7% vs 12.3%, respectively; *P* = 0.03), including discontinuations due to renal impairment (0.7% vs 1.4%, respectively; *P* = 0.002) [8]. Conversely, with less serious side effects, it is important to attempt to manage therapy before adjusting sacubitril/valsartan dose. In a subgroup analysis of PARADIGM-HF, dose reductions with sacubitril/valsartan and enalapril were associated with increased risk of cardiovascular death or hospitalizations for HF (HR 2.5; 95% CI 2.2–2.7) [32]. Furthermore, in our clinical experience, patients often feel well despite hypotension identified on assessment of vital signs; therefore, in the setting of clinical improvement, dose adjustment of other therapies should be considered first. Hypotension may also be managed by counseling patients to take medications at bedtime or by staggering medications if they are taken twice daily. Generally, we found that side effects of sacubitril/valsartan usually resolve within 14 days; therefore, it is important to follow up with patients every 2 weeks during uptitration. It can also be useful to have patients keep a BP diary, checking their BP measurements at the same time each day and when experiencing symptoms consistent with hypotension, and to educate them to call if their systolic BP (SBP) drops to < 90 mmHg or if they are experiencing dizziness, lightheadedness, or syncope.

If side effects persist, dose reduction of sacubitril/valsartan should be considered. Dose reductions are preferable to discontinuation of sacubitril/valsartan, because patients treated with lower-dose sacubitril/valsartan experienced reduced risk of cardiovascular death or hospitalization compared with patients treated with lower-dose enalapril (HR 0.80; 95% CI 0.70–0.93) that was similar to those who did not receive dose reductions (HR 0.79, 95% CI 0.71–0.88; *P* < 0.001) [32]. Re-uptitration should be attempted 1–2 weeks following resolution of side effects. Many patients treated with sacubitril/valsartan in PARADIGM-HF were successfully re-uptitrated following dose reduction. Regarding hypotension, a significantly higher proportion of patients treated with sacubitril/valsartan were successfully re-uptitrated versus enalapril (36% vs 27%; *P* = 0.026) [32].

In May 2016, Novartis established the FortiHFy global clinical program, which includes more than 40 active and planned studies to collect additional data on symptom reduction, efficacy, safety, QOL, and real-world evidence with sacubitril/valsartan [49]. Data collected from these studies may address some concerns associated with clinical use of sacubitril/valsartan.

## Suggested treatment sequence scheme

Successful implementation of sacubitril/valsartan in our practices required us to become comfortable prescribing sacubitril/valsartan, receive approval from our institutions, and maintain a good rapport with patients. We have developed a treatment scheme to guide clinicians to achieve success with sacubitril/valsartan use. This strategy, divided into three waves, can facilitate implementation of sacubitril/valsartan into cardiology clinics and the inpatient setting.

### Wave 1

Clinicians should begin implementation of sacubitril/valsartan by replacing ACEI/ARB therapy (with 36-h washout period) in stable outpatients with NYHA class II/III HFrEF, as recommended in ACC/AHA/HFSA HF management guidelines [2, 3]. When selecting initial patients to prescribe sacubitril/valsartan, clinicians should ensure their characteristics be similar to individuals in PARADIGM-HF [8] and consistent with the product label and clinical guidelines [2, 3, 10]. This includes NYHA class II HFrEF, SBP ≥ 100 mmHg, adequate kidney function, normal potassium levels, eGFR ≥ 30 mL/min/1.73 m^2^, and no history of angioedema [8, 10]. In this wave, patients should already be receiving a beta-blocker and ACEI/ARB [2, 3]. Treating patients with fewer symptoms and complications can help minimize risks while clinicians become accustomed to titrating doses and monitoring for side effects with sacubitril/valsartan.

### Wave 2

Once clinicians become more comfortable with monitoring sacubitril/valsartan, they may consider prescribing it to select patients hospitalized for acute HF before discharge. However, until August 2018, no data have demonstrated the safety and efficacy of initiating sacubitril/valsartan before acute HF discharge. This evidence gap was reflected by an implementation rate of 2.3% for sacubitril/valsartan before hospital discharge that was recently reported from a registry of HF admissions in US hospitals [50]. A separate analysis of the same registry observed that among 28,932 hospitalizations for HFrEF, 20,083 (69%) involved patients who met FDA labeling requirements for sacubitril/valsartan initiation and 11,018 (38%) involved patients meeting the stricter PARADIGM-HF inclusion criteria [51]. Thus, many patients may benefit from sacubitril/valsartan initiation at discharge. Results of the Comparison of Pre- and Post-Discharge Treatment Initiation With LCZ696 in Heart Failure Patients With Reduced Ejection-Fraction Hospitalized for an Acute Decompensation Event (TRANSITION; NCT02661217) trial presented at the European Society of Cardiology demonstrated that sacubitril/valsartan can be safely initiated after hemodynamic stabilization and prior to acute HF discharge [6]. In addition, the Comparison of Sacubitril/Valsartan Versus Enalapril on Effect on NT-proBNP in Patients Stabilized From an Acute Heart Failure Episode (PIONEER-HF; NCT02554890) trial will further evaluate this strategy [52].

Experienced clinicians with strong support staffs and follow-up protocols could initiate sacubitril/valsartan at hospital discharge with the goal of improving transitions of care and decreasing risk of readmission. Only patients appropriately treated for acute HF should be initiated on therapy with sacubitril/valsartan before discharge, because acute HF is not an indication for therapy and was an exclusion criterion of PARADIGM-HF [8]. Alternatively, switching ARNI therapy candidates from preadmission ACEI/ARB therapy to valsartan at discharge may be considered to facilitate outpatient initiation of sacubitril/valsartan. Once this wave is implemented, clinicians will learn to navigate the obstacles associated with monitoring sacubitril/valsartan through transitions of care.

### Wave 3

In this wave, clinicians may begin to use sacubitril/valsartan therapy to treat additional, informed patients willing to try a new drug. Clinicians can also consider switching treatment from ACEI or ARB to ARNI therapy in NYHA class IV HFrEF, as approved by the FDA [10] but not yet recommended in HF guidelines [3], recognizing that these patients were not well represented in PARADIGM-HF. During PARADIGM-HF’s run-in period, ~ 20% of patients failed to reach randomization [8]. Features associated with failure to reach randomization included renal dysfunction, elevated natriuretic peptides, ischemic etiology, and low SBP [53]. It remains unclear if patients with advanced HF—particularly those with hypotension, renal dysfunction, or markedly elevated natriuretic peptide levels—will be able to benefit from and tolerate sacubitril/valsartan. The ongoing Entresto (LCZ696) In Advanced HF (HFN-LIFE; NCT02816736) trial will provide more evidence that may support implementation of this wave [52].

Recognizing that there is no currently available published trial evidence to support the use of sacubitril/valsartan therapy in patients with NYHA class IV HFrEF, these patients should be closely monitored. Patients naive to ACEI/ARB therapy may be treated with sacubitril/valsartan if they meet eligibility requirements, recognizing that this group was not studied in PARADIGM-HF [52]. However, it is important to note that the TRANSITION trial enrolled a substantial number of patients with new-onset HFrEF (29%) and patients who were ACEI/ARB naïve (24%) [6]. At this point, clinicians should feel confident caring for all patients who may benefit from sacubitril/valsartan.

## Summary

ACEIs/ARBs were the standard of care for decades; however, sacubitril/valsartan has a unique MOA, in which synergistic effects of neprilysin inhibition with angiotensin receptor blockade improves efficacy. PARADIGM-HF was one of the largest clinical trials ever conducted in HF and demonstrated significant (20%) reduction in the risk of death from cardiovascular causes or hospitalization for HF with sacubitril/valsartan versus enalapril. Further analyses of PARADIGM-HF have identified additional benefits, including reductions in 30-day and 60-day hospital readmission rates and prevention of clinical progression in surviving patients. Based on these improved outcomes, it is necessary to make a paradigm shift in clinical practice to overcome obstacles to timely implementation of this lifesaving therapy. Barriers to the uptake of sacubitril/valsartan can be addressed with education, as evidence supporting its benefits continues to grow. According to US, European Union, and Canadian HF guidelines and emerging data, it is beneficial to implement sacubitril/valsartan in eligible patients to provide further reductions in mortality. Finally, appropriate and timely use of sacubitril/valsartan has the potential to significantly improve global and public health.
